# Effect of internet-based vs. in-person multimodal interventions on patients with mild to moderate Alzheimer’s disease: a randomized, cross-over, open-label trial

**DOI:** 10.3389/fpubh.2023.1203201

**Published:** 2023-07-07

**Authors:** Young Hee Jung, Sang-Cheol Park, Jee Hee Lee, Myong Jong Kim, Seunghoon Lee, Su Jin Chung, Ji Yeon Moon, Young Hi Choi, Jieun Ju, Hyun Jeong Han, So Young Lee

**Affiliations:** ^1^Department of Neurology, Myongji Hospital, Hanyang University College of Medicine, Goyang, Republic of Korea; ^2^Artificial Intelligence and Robotics Laboratory, Myongji Hospital, Goyang, Republic of Korea; ^3^Department of Public Health and Healthcare Service, Myongji Hospital, Hanyang University College of Medicine, Goyang, Republic of Korea; ^4^Center for Arts and Healing, Myongji Hospital, Hanyang University College of Medicine, Goyang, Republic of Korea; ^5^Department of Psychiatry, Myongji Hospital, Hanyang University College of Medicine, Goyang, Republic of Korea; ^6^Cheongpungho Geriatric Hospital, Jecheon, Republic of Korea

**Keywords:** Alzheimer’s disease, in-person, internet-based, art therapy, music therapy, multimodal intervention

## Abstract

**Objective:**

We aimed to investigate the effect of internet-based and in-person cognitive interventions on cognition, mood, and activities of daily living (ADL) on patients with mild to moderate Alzheimer’s disease (AD) and examine whether internet-based intervention is as effective as the in-person intervention.

**Methods:**

We recruited 52 patients with probable mild AD, of whom 42 completed the trial. We randomly divided participants into intervention and control groups at a 1:1 ratio and statistically compared the neuropsychological test results of the two groups. In addition, patients in the intervention group were randomly assigned to a 4 weeks internet-based or in-person intervention, with subsequent crossover to the other group for 4 weeks. We statistically analyzed and compared the neuropsychological test scores between internet-based and in-person interventions.

**Results:**

Compared with the control group, the intervention group (internet-based and in-person) showed significantly improved profile in cognition (*p* < 0.001), depression (*p* < 0.001), anxiety (*p* < 0.001) and ADL (*p* < 0.001). In addition, the effect of the internet-based intervention on cognition (*p* = 0.918) and depression (*p* = 0.282) was not significantly different from that of the in-person intervention. However, in the Beck anxiety inventory (*p* = 0.009) and Seoul instrumental activity of daily living (*p* = 0.023), in-person intervention was more effective than internet-based intervention.

**Conclusion:**

This study suggests that both types of cognitive intervention (in-person and internet-based) may be viable supplementary treatments along with approved pharmacological therapy. In terms of anxiety and ADL, the effect of the in-person interventions may be more effective than the-internet based interventions.

## Introduction

Alzheimer’s disease (AD), a prevalent form of dementia, represent a growing burden in an aging society (1). Currently AD affects around 40 million adults worldwide. Pharmacological treatment is used to slow the progression of the disease. However, pharmacological treatment alone cannot effectively delay the impairment, and multimodal treatment is necessary. Various studies have highlighted the positive impact cognitive interventions have on cognition in older adults with or without cognitive decline (2–4). Likewise, our previous studies demonstrated that the in-person multimodal intervention including cognitive training, music therapy and art therapy improves cognition, activities of daily living (ADL), and mood in mild to moderate AD (5, 6). Moreover, some studies found that art and music therapy can significantly alleviate depression and anxiety in patients with dementia (7–10). This findings underscore the potential benefits of non-pharmacological multimodal interventions, including cognitive training, art therapy, and music therapy, as good supplementary options to preserve cognitive function in patients with cognitive impairment.

The coronavirus disease 2019 (COVID-19) pandemic has challenged the in-person delivery of cognitive interventions to dementia patients (11). Older patients with dementia may avoid medical facilities or may be required to self-isolate due to lockdowns, illnesses, or fear of exposure to the virus (12, 13). Furthermore, cognitive intervention programs are limited due to money, time, and space requirements in real clinical settings. Therefore, there is an emerging need for remote cognitive training for AD. The COVID-19 pandemic has accelerated mobile health applications and telemedicine, and popularized teleconferencing technology (14). Additionally, information and communication technology (ICT) intervention involves utilizing tools such as the internet, handheld digital devices, computer terminals, and cellular phones (2, 15–18). The internet based intervention is different from mobile-based in that internet based intervention is based on the real time interaction with therapist. Internet-based video conferencing is more akin to in-person interventions as therapists can interact with and motivate participants, a feature not typically present in mobile-based interventions. Additionally, the internet-based intervention using Zoom allows for group interactions with multiple participants, which is unlike the mobile phone-based intervention (16). A recent studies indicated that cognitive interventions using ICT had a statistically significant positive impact on cognitive function when compared with various control groups (19). However, the effectiveness of the internet-based interventions compared to the in-person interventions has not been extensively explored. In the present study, we aimed to investigate the effect of the internet-based interventions on cognition, mood, and ADL on patients with mild to moderate AD, and examine whether it is as effective as in-person cognitive interventions.

## Method

### Participants

We registered the data of 52 patients at Myongji Hospital in Goyang, Republic of Korea, and Cheongpungho Geriatric Hospital from September to December 2021. All patients underwent a detailed medical history taking, neurological examinations, neuroimaging (brain computed tomography or magnetic resonance imaging), and blood and neuropsychological tests, and met the criteria of probable AD according to the National Institute of Neurological and Communicative Disorders and Stroke-Alzheimer Disease and Related Disorders Association (NINCDS-ADRDA) (20). Participants who had mild to moderate AD, which is a rating that corresponds to 0.5–1 in the Korean version of clinical dementia rating (CDR), were included in the study.

The exclusion criteria were the presence of metabolic diseases that could affect cognitive function (hyperthyroidism, vitamin B12 or folic acid deficiency, chronic renal failure, uncontrolled diabetes, and hepatic failure), chronic alcoholism, and a history of stroke, seizure, and brain surgery. Furthermore, patients who met the Diagnostic and Statistical Manual of Mental Disorders (Fifth edition) for psychotic or mood disorders such as schizophrenia or major depressive disorder were also excluded (9).

### Study design

**Figure 1:**
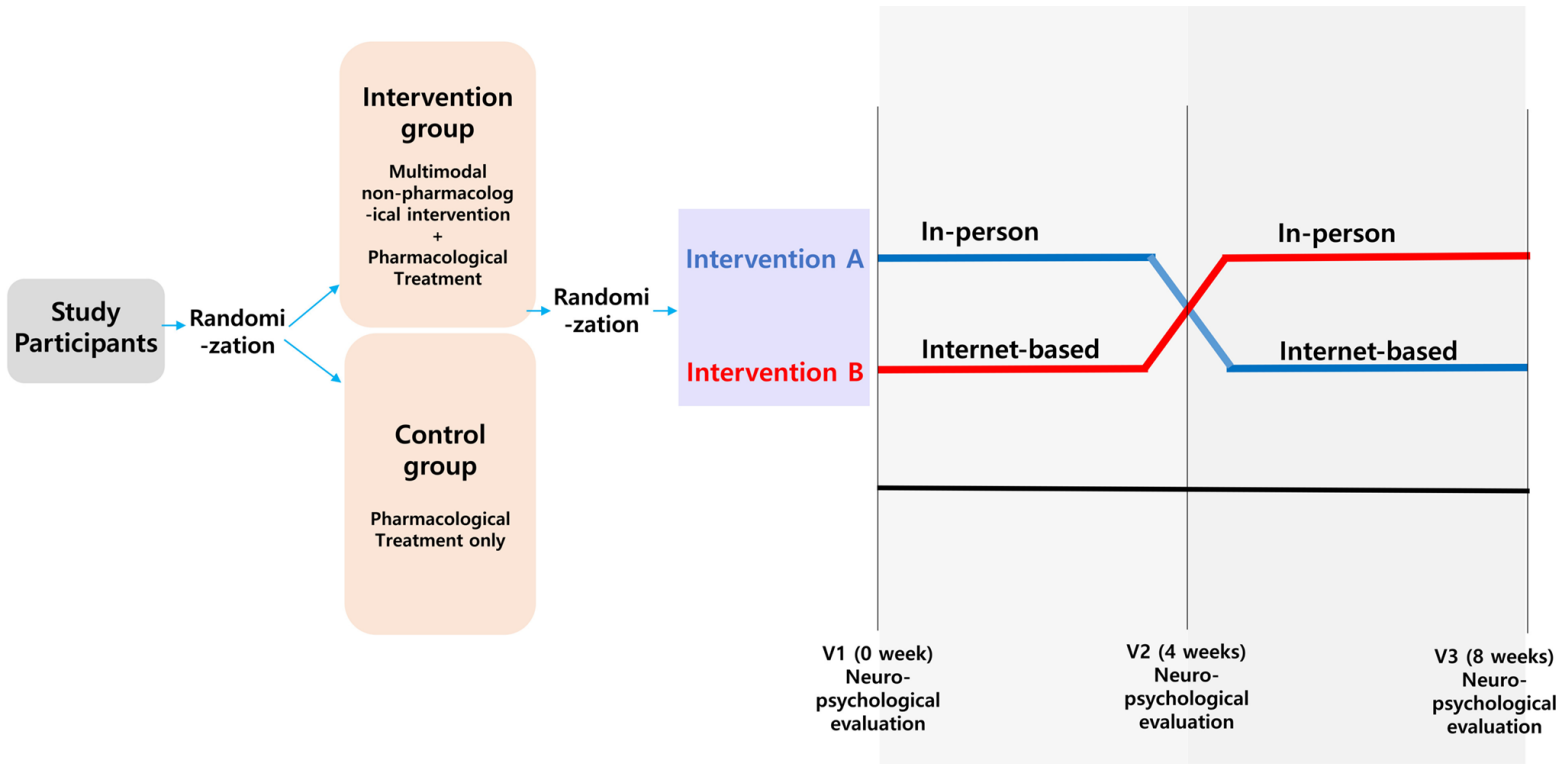
Study design. We randomly divided participants into intervention and control groups at a 1:1 ratio. In addition, patients in the intervention group were randomly assigned to a 4 weeks internet-based or in-person intervention, with subsequent crossover to the other group for 4 weeks.

Fifty-two eligible participants were randomized and allocated into the control (only pharmacological treatment, *n* = 26) and intervention groups (both non-pharmacological intervention and pharmacological treatment, *n* = 26) (Supplementary Figure S1). The intervention group was further classified into two groups, and intervention A group (*n* = 13) underwent an intervention over Zoom videoconferencing during a 4 weeks period and then an in-person intervention during a 4 weeks period. Intervention B (*n* = 13) group underwent an in-person intervention during a 4 weeks period, followed by the internet-based intervention (Zoom videoconferencing) during the remaining 4 weeks period (Figure 1). Both the control and intervention groups maintained the same doses of acetylcholine esterase inhibitor, N-methyl-D-aspartate receptor (NMDA) antagonist, selective serotonin reuptake inhibitor (SSRI), and benzodiazepine during the study period. All participants underwent a neuropsychological test at the fourth and eighth weeks. Participants who withdrew the consent were excluded according to approved protocols and guidelines of institutional review board of Myongji Hospital.

### Randomization

Using covariate-constrained randomization in the cvcrand package, the participants were randomized and allocated to the intervention and control groups. In addition, the participants in the case groups were randomized and allocated to intervention A and intervention B groups. When randomized, age, sex, and baseline CDR were controlled for.

### In-person and internet-based cognitive intervention programs

A nonpharmacological treatment program was established, incorporating elements of cognitive, music, and art therapies. This consisted of a 1 hour session (30 min each for 32 sessions), split evenly between cognitive training, and either music therapy or art therapy. Cognitive training was provided twice a week, while music and art therapies were conducted once a week. Cognitive training was provided twice a week, and music and art therapy were provided once a week. An internet-based multimodal intervention was delivered over Zoom.

Cognitive training comprised 16 sessions of 30 min each conducted over 8 weeks (twice a week). Out of these 16 sessions, eight were conducted via the internet and the other eight in person. Each session consisted of four stages: introductory activity, brain health lifestyle education, main activity, and finishing the activity. The primary activities included five domains: memory, attention, visuospatial function, frontal-executive function, and language and related functions.

Music therapy included eight 30 min sessions over 8 weeks (once a week), with half of these sessions conducted over Zoom and the rest in person. Each session was structured into three stages: introductory activity (5 min), main activity (20 min), and finishing activity (5 min). In the therapeutic song singing activity, the participants had time to listen to or sing all the prepared songs together, share feelings, and discuss the meaning of the lyrics. Afterward, each section was divided into groups and solos, and each group member sang in solo and proceeded in a solo-tutti structure performed in the chorus. In addition, therapeutic singing included the writing of song lyrics by group members. In the instrumental activity, participants were expected to improvise their rhythm. After presenting a variety of rhythmic instruments, participants selected their favorite instrument, added their interpretation of the music, and improvised the rhythm presented to them on the therapist’s piano. As participants grew comfortable with their instruments, they began to recognize their roles in the solo-chorus structure, preparing themselves for expression when performing with the rest of the group. The activity involved participants trying to play melody instruments like colored handbells by reading color scores.

Art therapy consisted of eight 30 min sessions over 8 weeks (once a week), evenly split between the internet-based and in-person sessions. The content of the internet based art therapy sessions were interconnected with the in-person sessions, involving the four specific sessions: “self-introduction,” (to make name tags), “Henri Matisse” (after appreciating a painting by Henri Matisse, to put the prepared color pieces together, and to make a single picture), “my hometown” (color the letters mandala and complete it), and “memory box” (to make a panorama that expresses the journey of life). The in-person art therapy sessions included the following activities: “self-introduction,” (to make name tags), “Henri Matisse” (after appreciating a painting by Henri Matisse, to carve them, and color each piece by individuals, and to take a picture and share the work by the therapist online), “my home town” (quizzes on my hometown online), and “memory box” [to make trees symbolize old age (present stage) by decorating it with pictures of one’s face and wishes, and share it online].

### Neuropsychological tests

The Seoul Neuropsychological Screening Battery (21, 22), which is a structured cognitive function assessment tool for evaluating each domain of cognitive function, was used as a basic examination. In this section, the Korean versions of the mini-mental status examination (K-MMSE) (23) and CDR (24) were performed. The red and blue forms of the K-MMSE were alternatively used to minimize the learning effect. Additionally, a Korean dementia screening questionnaire-cognition (KDSQ-C) was administered to the patients to evaluate their cognitive function (25). Before and after patients underwent Zoom and in-person intervention, the long form of the geriatric depression scale (GDS) (26), Beck anxiety inventory (BAI) (27), and Seoul-instrumental activities of daily living (S-IADL) (28) were used to assess the emotions and ADL of each patient. The cutoff values were as follows: long form of GDS, 18 out of 30; BAI, 22 out of 63; S-IADL, 8 out of 45; and KDSQ-C, 6 out of 15. A follow-up cognitive assessment was performed within 4 months of the study.

### Assessment of the level of satisfaction

The level of satisfaction was assessed using the following questions: (1) Was the Centenarian’s Good Memory Program satisfactory? (2) Was the schedule of the Centenarian’s Good Memory Program satisfactory? (3) Were the space and facilities of the Centenarian’s Good Memory Program satisfactory? (4) Was the Centenarian’s Good Memory Program helpful in the prevention of dementia in daily life? (5) Would you recommend participating in the Centenarian’s Good-Memory Program to your acquaintances? (6) Do you want to participate in the Centenarian’s Good Memory Program? Responses to each question were graded from 1 (*not at all satisfied*) to 5 (*very satisfied*), and the total score was 30.

### Statistical analysis

Demographic characteristics between the control and intervention groups were compared using a two-sample *t*-test for continuous variables and a chi-squared test of independence for categorical variables. Next, the changes in cognition, ADL, and mood before and after the integrated cognitive therapy were analyzed using analyses of covariance (ANCOVAs) to evaluate the effectiveness of the intervention with covariates (educational year and CDR-SOB) to control for confounding factors. We excluded age and sex, which were used for randomization, from the covariates in the analysis. Statistical significance is indicated by *p* ≤ 0.05. All analyses were performed using SAS^®^ 9.4.

## Results

### Participants’ flow, adherence to interventions, and baseline characteristics

Fifty-six patients were included in this study. Fifty-two participants were randomized and assigned in a 1:1 ratio to the intervention and control groups The intervention group was also divided into two subgroups, intervention A and intervention B, through random allocation: (i) intervention A group underwent an internet-based intervention for 4 weeks, followed by in-person therapy for 4 weeks; (ii) intervention B group received the opposite sequence of intervention. In the control group, five participants were excluded according to approved protocols and guidelines of institutional review board because the patients withdrew the consents. Three participants in intervention A group and two in intervention B group discontinued the intervention and were excluded (Supplementary Figure S1) with the same reason. The mean number of sessions that each patient participated in was 15.5 ± 0.8 out of 16. Baseline characteristics were balanced between the intervention and control groups as there were no significant differences in age, sex, education, cognitive scores, and depression and anxiety scores (Table 1).

**Table 1:** Demographic and clinical characteristics.

	Overall (*n* = 42)	Control (*n* = 21)	Intervention (*n* = 21)	*p*-value
Female	66.7 (28)	66.7 (14)	66.7 (14)	1.000
Age	77.4 ± 8.4	75.4 ± 8.4	79.3 ± 8.1	0.773
Education year	8.4 ± 5.1	8.0 ± 5.0	8.8 ± 5.2	0.994
Comorbidities				
Hypertension	59.5 (25)	69.1 (13)	57.1 (12)	0.753
Diabetes mellitus	23.8 (10)	33.3 (7)	14.3 (3)	0.147
Dyslipidemia	40.5 (17)	38.1 (8)	42.9 (9)	0.753
Cardiac disease	9.5 (4)	9.5 (2)	9.5 (2)	1.000
Stroke	4.8 (2)	9.5 (2)	0 (0)	0.147
Medication				
Acetylcholine esterase inhibitor	64.3 (27)	66.7 (14)	61.9 (13)	0.747
NMDA antagonist	7.1 (3)	4.8 (1)	9.5 (2)	0.549
SSRI	26.2 (11)	42.9 (9)	9.5 (2)	0.014
Benzodiazepine	23.8 (10)	23.8 (5)	23.8 (5)	1.000
Baseline score				
MMSE	21.2 ± 4.9	20.8 ± 4.3	21.9 ± 5.5	0.575
GdepS	13.8 ± 10.7	15.0 ± 10.4	12.6 ± 11.0	0.425
BAI	14.4 ± 17.0	17.4 ± 19.4	11.4 ± 14.1	0.261
SIADL	17.1 ± 12.0	18.8 ± 11.6	15.5 ± 12.3	0.374
CDR	0.750 ± 0.253	0.738 ± 0.256	0.762 ± 0.256	0.765
CDR = 0.5, % (*n*)	50 (21)	47.6 (10)	52.4 (11)	
CDR = 1	50 (21)	52.4 (11)	47.6 (10)	

NMDA, N-methyl-D-aspartate receptor; SSRI, selective serotonin reuptake inhibitor; MMSE, mini-mental state examination; GdepS, geriatric depression scale; BAI, Beck anxiety inventory; SIADL, Seoul instrumental activity of daily living; CDR, clinical dementia rating.

### Comparison of the score of neuropsychological tests between control and intervention groups

**Figure 2:**
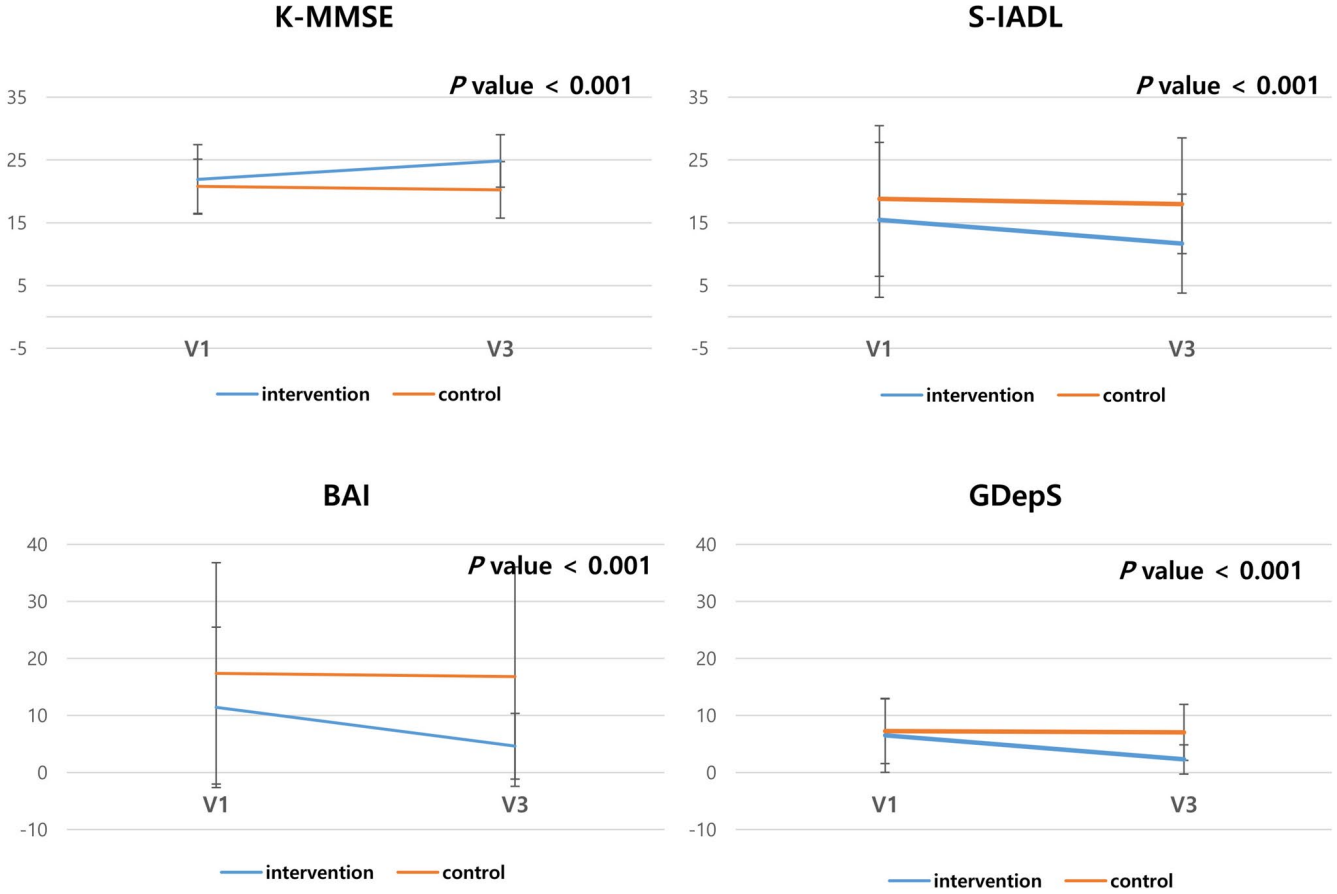
Comparison of the neuropsychological test results of the control and intervention groups. The scores of K-MMSE, KDSQ, GdepS, BAI and SIADL after 8 weeks was compared with baseline scores.

When comparing the effect of non-pharmacological interventions [internet-based intervention (4 weeks) and in-person interventions (4 weeks)] with the control group (pharmacological treatment only for 8 weeks) after controlling for CDR-SOB and education year using ANCOVA, the K-MMSE (*p* < 0.001) and KDSQ scores (*p* < 0.001) improved in the intervention group. In terms of mood, the GdepS (*p* < 0.001) and BAI scores (*p* < 0.001) improved in the intervention group. Similarly, the S-IADL score (*p* < 0.001) also significantly improved in the intervention group (Table 2 and Figure 2).

**Table 2:** Comparison of the neuropsychological test results before and after intervention.

	Control (*n* = 21)		Intervention (*n* = 21)		*F*	*p*-value^*^
	0 week	8 weeks	0 week	8 weeks		
K-MMSE	20.8 ± 4.3	20.2 ± 4.5	21.9 ± 5.5	24.9 ± 4.2	25.55	<0.001
KDSQ	17.3 ± 7.3	16.3 ± 7.1	12.5 ± 6.9	8.8 ± 6.1	19.2	<0.001
GdepS	15.0 ± 10.4	14.4 ± 9.5	12.6 ± 11.0	4.4 ± 4.0	22.2	<0.001
BAI	17.4 ± 19.4	16.8 ± 19.2	11.4 ± 14.1	4.6 ± 5.8	95.4	<0.001
SIADL	18.8 ± 11.6	18.0 ± 10.6	15.5 ± 12.3	11.7 ± 7.9	43.5	<0.001

MMSE, mini-mental status examination; KDSQ, Korean dementia screening questionnaire; GdepS, geriatric depression scale; BAI, Beck anxiety inventory; SIADL, Seoul instrumental activities of daily living. Post-intervention scores were compared using analysis of covariance (ANCOVA) after adjusting for the baseline education year and clinical dementia rating-sum of boxes (CDR-SOB).

### Comparison of neuropsychological profiles and the level of satisfaction between the internet-based and in-person intervention periods

**Figure 3:**
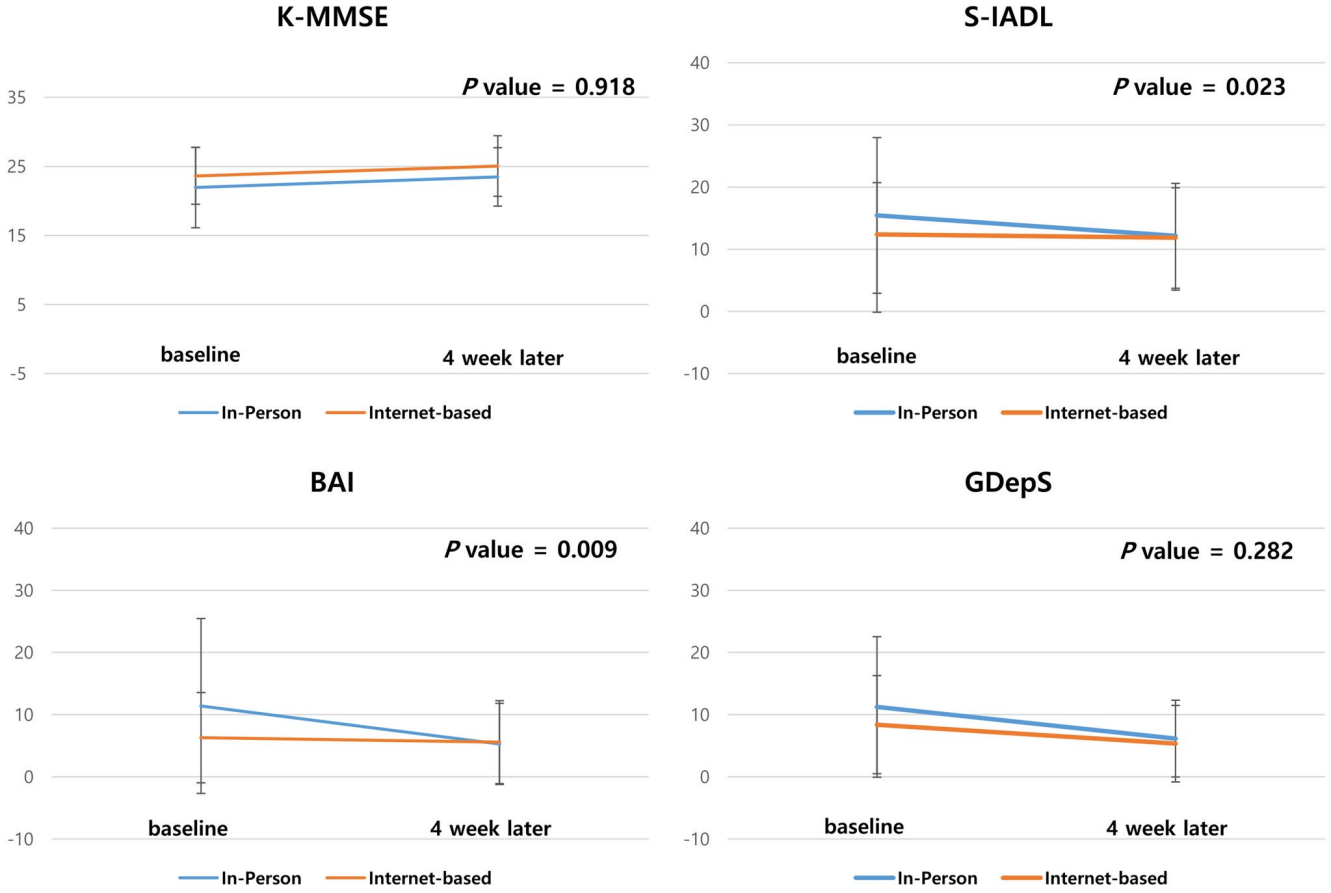
Comparison of the score of neuropsychological tests of the Zoom and in-person intervention periods. The scores difference of K-MMSE, KDSQ, GdepS, BAI, SIADL and level of satisfaction during the Zoom intervention period were compared with the score difference during the in-person intervention period.

For the same participants, we compared the difference in scores during the internet-based and in-person intervention period. The score difference on the internet-based intervention period was not significantly different from that of the in-person intervention period in the MMSE (*p* = 0.918) and GDepS (*p* = 0.282). The KDSQ-C (*p* = 0.026), BAI (*p* = 0.009), and SIADL (*p* = 0.023) scores improved more after the in-person than the internet-based intervention (Table 3 and Figure 3).

**Table 3:** Comparison of the neuropsychological test results according to the type of cognitive intervention (*n* = 21).

	In-person intervention period		Internet-based intervention period		*F*	*p*-value^*^
	Baseline	4 weeks later	Baseline	4 weeks later		
K-MMSE	22.0 ± 5.8	23.5 ± 4.2	23.6 ± 4.1	25.0 ± 4.4	5.65	0.918
KDSQ	13.0 ± 7.2	9.4 ± 6.2	9.5 ± 5.7	9.4 ± 6.2	5.4	0.026
GdepS	11.2 ± 11.3	6.1 ± 6.2	8.4 ± 7.9	5.3 ± 6.2	1.2	0.282
BAI	11.4 ± 14.1	5.3 ± 6.5	6.3 ± 7.3	5.6 ± 6.7	7.5	0.009
SIADL	15.4 ± 12.5	12.1 ± 8.4	12.4 ± 8.3	11.9 ± 8.0	0.01	0.023

MMSE, mini-mental status examination; KDSQ, Korean dementia screening questionnaire; GdepS, geriatric depression scale; BAI, Beck anxiety inventory; SIADL, Seoul instrumental activities of daily living. Post-intervention scores were compared using analysis of covariance (ANCOVA) after adjusting for the baseline education year and clinical dementia rating-sum of boxes (CDR-SOB).

In addition, no significant difference was observed in the level of satisfaction between the internet-based and in-person interventions (24.7 vs. 24.5, *p* = 0.940) (Supplementary Figure S2).

## Discussion

The present study was a randomized controlled trial (RCT) that investigated the effect of the internet-based and in-person interventions on cognitive function, mood, and ADL of 42 patients with probable AD according to the NINCDS-ADRDA criteria. This study suggests that the multimodal intervention, in conjunction with pharmacological treatment, was more effective in improving cognitive function, mood, and ADL than pharmacological treatment alone which is compatible with previous studies (4, 16, 19, 29). Moreover, the effect of the internet-based intervention may not significantly different from that of the in-person intervention in improving cognition and depression; however, there was significant difference between the in-person intervention and the internet-based intervention in improving anxiety and ADL. In addition, the level of satisfaction with the internet-based intervention period was not significantly different from that of the in-person intervention.

Previous studies have shown that non-pharmacological interventions, including cognitive training, affect cognition in older adults (30–32). Recently, with the development of technology and the COVID-19 pandemic, the importance of remote delivery of non-pharmacological interventions is emerging (11). A few clinical trials that used smartphone applications as cognitive training tools showed effects on cognition in older individuals without dementia (2, 15). There is previous literature discussing the use of in-home video telehealth for delivering interventions to individuals with dementia. Burton and O’Connell (33) examined the use of cognitive rehabilitation for goal setting via telehealth videoconferencing in six people with subjective cognitive impairment, mild cognitive impairment, or Alzheimer’s disease and concluded that the intervention could be feasibly delivered Jelcic et al. (34) used Skype as a platform to deliver their cognitive rehabilitation program that consisted of lexical tasks aimed at enhancing semantic verbal processing in 27 people with Alzheimer’s disease. They also concluded that it was feasible to deliver their program, which might improve global cognitive performance. Our RCT is distinguishable from previous studies for the following reasons: first this study demonstrated that ICT-based interventions, such as Zoom, are feasible for delivering multimodal interventions. ICT-based interventions utilize various tools such as the internet, mobile phones, and handheld devices. Among them, the internet-based methods, particularly those using platforms like Zoom, offer real-time interaction with therapists, similar to the in-person sessions, and uniquely allow for group participation, a feature typically absent in mobile-based interventions. Second, art and music therapy programs as well as cognitive training were adapted to an online environment in this study. Third, this study compared the efficacy of traditional in-person interventions with that of internet-based interventions, demonstrating that internet-based interventions are as effective as in-person interventions in treating depression and improving cognition. Therefore, it shows the promise of online intervention in the real world, in that, it can be provided in situations such as lockdowns due to the COVID-19 pandemic or mobility problems due to gait disturbance, regardless of where people are located.

First, cognitive intervention was more effective in improving the K-MMSE, S-IADL, GdepS, and BAI scores in the case group than in the control group with only pharmacological treatment. In terms of cognition, because of the practice effect of repeated tests, we double-checked the subjective change in the cognitive function to be identified to caregivers using the KDSQ-C. The KDSQ-C score of the intervention group significantly improved compared to that of the control group. Considering that existing pharmacological treatment has only a limited effect on ADL, it is noteworthy that it added evidence of the effect of cognitive intervention on ADL (29). In addition, consistent with previous studies, multimodal intervention more effectively alleviated depression and anxiety in patients with mild to moderate AD than pharmacological treatment in the control group (29, 35).

Second, when the effect of the internet-based intervention was compared with that of the in-person intervention among the same patients, the effects on cognition and depression were not significantly different between the two interventions. However, the internet-based intervention may be as effective as in-person intervention for improving anxiety and ADL. Regarding depression, the internet-based intervention might be as effective as in-person interventions, which is consistent with a previous study that found that group intervention relieves loneliness and depression among older people during the COVID-19 pandemic (36, 37). Additionally, more research is needed to develop the internet-based interventions that can effectively improve anxiety and ADL.

This RCT demonstrated that non-pharmacological multimodal interventions (internet-based and in-person interventions) improve cognitive function, ADL, and mood in patients with AD compared to the control group (pharmacological treatment only). In addition, the effect of the internet-based intervention on cognition and depression was not significantly different from that of the in-person intervention. Therefore, the internet-based intervention may be effective enough to be applied to patients with mild to moderate AD. However, our study has some limitations. First, it’s difficult to say that this study perfectly controlled the effects of the medication. There was no significant difference in the proportion of drug use between the two groups and we maintained the type and dosage of medication from the original drug treatment during the research period, although we could not completely stop medication and purely observe the effects of the intervention due to ethical considerations. Second, the washout period was not allocated between the two intervention periods, considering that each intervention period was only 4 weeks long, and the intervention may not be residual in the body, unlike pharmacologic treatment. Third, we did not use an amyloid biomarker to diagnose AD; therefore, diagnostic uncertainty remains unclear. Fourth, the sample size was relatively small, and the study period was not long. Particularly, due to the limited sample size in both intervention A and B group, it’s still uncertain whether the internet-based interventions is effective as the in-person interventions.

In conclusion, this study demonstrated that the intervention (in-person and internet-based) group showed improved cognition, ADL, and mood more than the control group in patients with mild to moderate AD. In terms of anxiety and ADL, the effect of the in-person interventions might be more effective than the-internet based interventions. Furthermore, this study suggested that both types of non-pharmacological interventions (in-person and internet-based interventions) may be viable supplementary treatments to be administered along with an approved pharmacological therapy.

## Data availability statement

The raw data supporting the conclusions of this article will be made available by the authors, without undue reservation.

## Ethics statement

The studies involving human participants were reviewed and approved by the institutional review board of Myongji Hospital (2021-05-009). The patients/participants provided their written informed consent to participate in this study.

## Author contributions

YJ and SYL conceived this study and wrote the draft. YJ, SYL, HH, JL MK, YC, SC, SL, and JJ collected and analyzed data. S-CP helped the data analysis. YJ and SYL revised the manuscript. All authors contributed to the articles and approved the submitted version.

## Funding

This Study was supported by the Newhorizon grant of Myongji Hospital (2013-07-01) and by a fund from the Korea Centers for Disease Control and Prevention (2023-ER1003-00).

## Conflict of interest

The authors declare that the research was conducted in the absence of any commercial or financial relationships that could be construed as a potential conflict of interest.

## Publisher’s note

All claims expressed in this article are solely those of the authors and do not necessarily represent those of their affiliated organizations, or those of the publisher, the editors and the reviewers. Any product that may be evaluated in this article, or claim that may be made by its manufacturer, is not guaranteed or endorsed by the publisher.

## References

[1] Wimo A Guerchet M Ali G-C Wu Y-T Prina AM Winblad B. The worldwide costs of dementia 2015 and comparisons with 2010. Alzheimers Dement. (2017) 13:1–7. doi: 10.1016/j.jalz.2016.07.150, PMID: 27583652 PMC5232417

[2] Hong YJ Lee J-H Choi EJ Han N Kim JE Park S-H. Efficacies of cognitive interventions in the elderly with subjective cognitive decline: a prospective, three-arm, controlled trial. J Clin Neurol. (2020) 16:304–13. doi: 10.3988/jcn.2020.16.2.304, PMID: 32319248 PMC7174106

[3] Jeong JH Na HR Choi SH Kim J Na DL Seo SW. Group-and home-based cognitive intervention for patients with mild cognitive impairment: a randomized controlled trial. Psychother Psychosom. (2016) 85:198–207. doi: 10.1159/000442261 27230861

[4] Carrion C Folkvord F Anastasiadou D Aymerich M. Cognitive therapy for dementia patients: a systematic review. Dement Geriatr Cogn Disord. (2018) 46:1–26. doi: 10.1159/000490851 30092585

[5] Jung YH Lee S Kim WJ Lee JH Kim MJ Han HJ. Effect of integrated cognitive intervention therapy in patients with mild to moderate Alzheimer’s disease. Dement Neurocogn Disord. (2020) 19:86–95. doi: 10.12779/dnd.2020.19.3.86, PMID: 32985148 PMC7521951

[6] Han HJ Son SJ Ha J Lee JH Kim SA Lee SY. The effect of group musical therapy on depression and activities on daily living in patients with cognitive decline. Dement Neurocogn Disord. (2014) 13:107–11. doi: 10.12779/dnd.2014.13.4.107

[7] Zhang Y Cai J An L Hui F Ren T Ma H. Does music therapy enhance behavioral and cognitive function in elderly dementia patients? A systematic review and meta-analysis. Ageing Res Rev. (2017) 35:1–11. doi: 10.1016/j.arr.2016.12.003 28025173

[8] Vink AC Zuidersma M Boersma F de Jonge P Zuidema SU JP S. The effect of music therapy compared with general recreational activities in reducing agitation in people with dementia: a randomised controlled trial. Int J Geriatr Psychiatry. (2013) 28:1031–8. doi: 10.1002/gps.3924 23280604

[9] Chancellor B Duncan A A C. Art therapy for Alzheimer's disease and other dementias. J Alzheimers Dis. (2014) 39:1–11. doi: 10.3233/JAD-131295 24121964

[10] Im ML Lee JI. Effects of art and music therapy on depression and cognitive function of the elderly. Technol Health Care. (2014) 22:453–8. doi: 10.3233/THC-140803, PMID: 24704654

[11] Armitage R Nellums LB. COVID-19 and the consequences of isolating the elderly. Lancet Public Health. (2020) 5:e256. doi: 10.1016/S2468-2667(20)30061-X, PMID: 32199471 PMC7104160

[12] Canevelli M Valletta M Blasi MT Remoli G Sarti G Nuti F. Facing dementia during the COVID-19 outbreak. J Am Geriatr Soc. (2020) 68:1673–6. doi: 10.1111/jgs.16644, PMID: 32516441 PMC7300919

[13] Manca R De Marco M Venneri A. The impact of COVID-19 infection and enforced prolonged social isolation on neuropsychiatric symptoms in older adults with and without dementia: a review. Front Psychiatry. (2020) 11:1086. doi: 10.3389/fpsyt.2020.585540 PMC7649825 33192732

[14] Moore AL Miller TM Ledbetter C. Remote vs. in-person delivery of LearningRx one-on-one cognitive training during the COVID-19 pandemic: a non-inferiority study. Front Psychol. (2021) 12:12. doi: 10.3389/fpsyg.2021.749898 PMC8586525 34777146

[15] Jang H Yeo M Cho J Kim S Chin J Kim HJ. Effects of smartphone application-based cognitive training at home on cognition in community-dwelling non-demented elderly individuals: a randomized controlled trial. Alzheimers Dement. (2021) 7:e12209. doi: 10.1002/trc2.12209 PMC8719348 35005202

[16] Cheung G Peri KJA. Challenges to dementia care during COVID-19: Innovations in remote delivery of group cognitive stimulation therapy. Aging Ment Health. (2021) 25:977–9. doi: 10.1080/13607863.2020.1789945 32631103

[17] Marcus BH Nigg CR Riebe D LH F. Interactive communication strategies: implications for population-based physical-activity promotion. Am J Prev Med. (2000) 19:121–6. doi: 10.1016/S0749-3797(00)00186-0, PMID: 10913903

[18] CR N. Technology’s influence on physical activity and exercise science: the present and the future. J Sport Exerc Psychol. (2003) 4:57–65. doi: 10.1016/S1469-0292(02)00017-1

[19] Jung A-R Kim D Park E-A Health P. Cognitive intervention using information and communication technology for older adults with mild cognitive impairment: a systematic review and meta-analysis. Int J Environ Res Public Health. (2021) 18:11535. doi: 10.3390/ijerph182111535, PMID: 34770049 PMC8583509

[20] McKhann GM Knopman DS Chertkow H Hyman BT Jack CR Jr Kawas CH. The diagnosis of dementia due to Alzheimer’s disease: recommendations from the National Institute on Aging-Alzheimer’s association workgroups on diagnostic guidelines for Alzheimer’s disease. Alzheimers Dement. (2011) 7:263–9. doi: 10.1016/j.jalz.2011.03.005, PMID: 21514250 PMC3312024

[21] Kang SH Park YH Lee D Kim JP Chin J Ahn Y. The cortical neuroanatomy related to specific neuropsychological deficits in Alzheimer’s continuum. Dement Neurocogn Disord. (2019) 18:77. doi: 10.12779/dnd.2019.18.3.77 31681443 PMC6819670

[22] Kang Y Na D Hahn S. Seoul neuropsychological screening battery. Incheon: Human Brain Research & Consulting Co. (2003).

[23] Han C Jo SA Jo I Kim E Park MH Kang Y. An adaptation of the Korean mini-mental state examination (K-MMSE) in elderly Koreans: demographic influence and population-based norms (the AGE study). Arch Gerontol Geriatr. (2008) 47:302–10. doi: 10.1016/j.archger.2007.08.012, PMID: 17936377

[24] Morris JC. The Clinical Dementia Rating (CDR): current version and scoring rules. Neurology. (1993) 43:2414–4. doi: 10.1212/wnl.43.11.2412-a 8232972

[25] Chey J. The development and validation of Korean dementia screening questionnaire (KDSQ). J Korean Neurol Assoc. (2002) 20:135–141.

[26] Yesavage JA Brink TL Rose TL Lum O Huang V Adey M. Development and validation of a geriatric depression screening scale: a preliminary report. J Psychiatr Res. (1982) 17:37–49. doi: 10.1016/0022-3956(82)90033-4, PMID: 7183759

[27] Beck AT Epstein N Brown G Steer RA. An inventory for measuring clinical anxiety: psychometric properties. J Consult Clin Psychol. (1988) 56:893–7. doi: 10.1037/0022-006X.56.6.893 3204199

[28] Ku H-M Kim J-H Kwon E-J Kim S-H Lee H-S Ko H-J. A study on the reliability and validity of Seoul-instrumental activities of daily living (S-IADL). J Korean Neuropsychiatr Assoc. (2004) 43:189–199.

[29] Chae HJ SH L. Effectiveness of online-based cognitive intervention in community-dwelling older adults with cognitive dysfunction: a systematic review and meta-analysis. Int J Geriatr Psychiatry. (2023) 38:e5853. doi: 10.1002/gps.5853, PMID: 36468299 PMC10107881

[30] Ngandu T Lehtisalo J Solomon A Levälahti E Ahtiluoto S Antikainen R. A 2 year multidomain intervention of diet, exercise, cognitive training, and vascular risk monitoring versus control to prevent cognitive decline in at-risk elderly people (FINGER): a randomised controlled trial. Lancet. (2015) 385:2255–63. doi: 10.1016/S0140-6736(15)60461-5 25771249

[31] Rosenberg A Ngandu T Rusanen M Antikainen R Bäckman L Havulinna S. Multidomain lifestyle intervention benefits a large elderly population at risk for cognitive decline and dementia regardless of baseline characteristics: the FINGER trial. Alzheimers Dement. (2018) 14:263–70. doi: 10.1016/j.jalz.2017.09.006, PMID: 29055814

[32] Simon SS Yokomizo JE Bottino CM. Cognitive intervention in amnestic mild cognitive impairment: a systematic review. Neurosci Biobehav Rev. (2012) 36:1163–78. doi: 10.1016/j.neubiorev.2012.01.007 22322184

[33] Burton RL O’Connell ME. Telehealth rehabilitation for cognitive impairment: randomized controlled feasibility trial. JMIR Res Protoc. (2018) 7:e9420. doi: 10.2196/resprot.9420, PMID: 29422453 PMC5824099

[34] Jelcic N Agostini M Meneghello F Bussè C Parise S Galano A. Feasibility and efficacy of cognitive telerehabilitation in early Alzheimer’s disease: a pilot study. Clin Interv Aging. (2014) 9:1605–11. doi: 10.2147/CIA.S68145, PMID: 25284993 PMC4181448

[35] Tay KW Subramaniam P Oei TPJP. Cognitive behavioural therapy can be effective in treating anxiety and depression in persons with dementia: a systematic review. Psychogeriatrics. (2019) 19:264–75. doi: 10.1111/psyg.12391, PMID: 30548731

[36] Shapira S Yeshua-Katz D Cohn-Schwartz E Aharonson-Daniel L Sarid O Clarfield AM. A pilot randomized controlled trial of a group intervention via zoom to relieve loneliness and depressive symptoms among older persons during the COVID-19 outbreak. Internet Interv. (2021) 24:100368. doi: 10.1016/j.invent.2021.100368, PMID: 33527072 PMC7839498

[37] Choi HK Lee SH. Trends and effectiveness of ICT interventions for the elderly to reduce loneliness: a systematic review. Healthcare. (2021) 9:293:293. doi: 10.3390/healthcare9030293 33800099 PMC8002106

